# Prospective Comparison of Local Anesthesia with General or Spinal Anesthesia in Patients Treated with Microscopic Varicocelectomy

**DOI:** 10.3390/jcm11216397

**Published:** 2022-10-28

**Authors:** Xiaobin Wang, Chunyu Pan, Jia Li, Yunhong Zhan, Gang Liu, Song Bai, Jun Chai, Liping Shan

**Affiliations:** 1 Center for Reproductive Medicine, Department of Obstetrics and Gynecology, Shengjing Hospital of China Medical University, Shenyang 110004, China; 2 Department of Urology, Shengjing Hospital of China Medical University, Shenyang 110004, China; 3 Department of Anesthesiology, Shengjing Hospital of China Medical University, Shenyang 110004, China

**Keywords:** local anesthesia, general or spinal anesthesia, microscopic varicocelectomy, varicocele

## Abstract

It is unclear whether local anesthesia (LA) is a viable and safe alternative to general anesthesia (GA) or spinal anesthesia (SA) for microscopic varicocelectomy. As a result, we designed a prospective trial to compare the pain relief, complications, and cost of LA with GA or SA in subinguinal microscopic varicocelectomy (MSV), using the propensity score matching method (PSM). This prospective study was conducted in a tertiary hospital from February 2021 to April 2022. Patients who underwent subinguinal MSV for varicocele were enrolled. The perioperative visual analog scale (VAS) scores, anesthesia-associated side effects, and cost data were recorded, and PSM analysis was performed. Finally, 354 patients were included, of whom 61.0% (216) were treated with LA, and 39.0% (138) underwent GA or LA. After PSM, the patients in the LA group exhibited lower VAS scores both three hours and one day after surgery, and a lower incidence of postoperative analgesic requirement; a lower ratio of patients who experienced anesthesia-associated side effects was also observed in the LA group, compared with the GA or SA group (all *p* < 0.001). The rate of perioperative satisfaction for patients was higher, the hospital stays and days to return to normal activity were shorter, and the cost was less in the LA group than in the patients in the GA or SA group (all *p* < 0.001). This prospective PSM cohort demonstrated that LA has the advantages of perioperative pain relief, reduced anesthesia-associated side effects, and cost, compared with GA or SA. It indicated that LA is an effective and safe technique for subinguinal MSV, and may guide clinical practice.

## 1. Introduction

Varicocele is characterized by abnormal dilatation of testicular veins in the pampiniform plexus, and is caused by impaired venous drainage [1]. It is a common genital abnormality that can progressively affect male fertility and testicular growth and lead to symptoms of scrotal pain and discomfort, even hypogonadism. Varicocele is predominantly left-sided, with an incidence of 95–99% [2]. Varicocele is present in almost 15% of the normal male population, 25% of men with poor semen quality, and 35–40% of infertile men [3].

Varicocelectomy is the most frequently performed procedure. The goal of varicocele treatment is to eliminate the dilatation of the pampiniform venous plexus and, hence, to obstruct the refluxing venous drainage to the testis [4]. Several techniques have been used, including microscopic surgery, laparoscopic surgery, interventional embolization, and open surgery [5]. The ideal varicocele treatment strategy should combine efficacy and safety, using the least invasive technique. For this reason, microscopic varicocelectomy (MSV) has gradually become the most preferred surgical option, due to its advantages of meticulous ligation of all the veins while sparing the arterial branches, lower postoperative recurrence rate, fewer complications, higher postoperative semen quality, and higher postoperative fertility rate [6,7,8]. MSV can be performed using either the inguinal or the subinguinal approach; the subinguinal approach involves the shallow spermatic cord and simple anatomic layers, which does not require the dissection of the external abdominal oblique muscle, and allows for the application of local anesthesia. This results in minor surgical trauma and a shorter recovery time [9].

Subinguinal MSV can be successfully performed under general anesthesia (GA), spinal anesthesia (SA), or local anesthesia (LA). The preferred anesthesia technique depends on several factors, including the procedure’s suitability for the patient, surgeon choice, patient acceptance, safety, perioperative pain control, the time to return to normal activity, the need for monitoring, and cost-effectiveness. LA is associated with reduced pain, decreased postoperative analgesic requirement, less need for postoperative care, lowered postoperative nausea and vomiting complications, a short anesthesia time and a short hospital stay, compared with GA [10,11]. Although many studies have compared LA and SA or GA for hernia repair, limited studies have compared them for varicocelectomy, and it is not clear whether LA is a suitable alternative to SA or GA for the procedure.

A retrospective study that included 3565 patients with varicocele demonstrated that inguinal MSV under LA (2% lidocaine) was superior to subinguinal MSV under GA or SA in operating duration and time to return to normal activity. However, no statistical difference was found in pain scores due to surgery [10]. An RCT study enrolled 60 patients who underwent subinguinal varicocelectomy and indicated that the visual analog scale (VAS) of pain under LA (2% prilocaine) was comparable with patients under SA, postoperatively. However, the mean dosage of injected diclofenac requirement was higher in the SA group compared with the LA group. The SA group also developed more postoperative complications, such as urinary retention, postspinal backache, headache, hypotension, and delayed mobilization in the postoperative period. Therefore, they considered LA an effective and safe alternative anesthetic method for subinguinal varicocelectomy [11].

Ropivacaine is a new long-acting local anesthetic. It produces cutaneous vasoconstriction that restricts systemic absorption of the drug and increases its local duration of action. Moreover, ropivacaine produces an anti-inflammatory effect that may further reduce the pain when administered locally. A mixture of equal volumes of 0.5% ropivacaine and 2% lidocaine has been widely used for LA among many diseases [12]. Lidocaine is believed to hasten the onset of the block, and ropivacaine provides a prolonged duration of the sensory block, extending to the postoperative period. To the best of our knowledge, no study has reported the effect of instilling a mixture of ropivacaine and lidocaine as a local anesthetic agent in MSV. The studies addressing this issue are rare, use retrospective designs, have small sample sizes, and the conclusions are debated. Therefore, we designed a prospective study to determine whether LA (a mixture of ropivacaine with lidocaine) was an acceptable alternative to GA or SA in MSV in terms of pain relief, complications, and cost, based on a large cohort using the propensity score matching method.

## 2. Methods

### 2.1. Study Design

**Figure 1:**
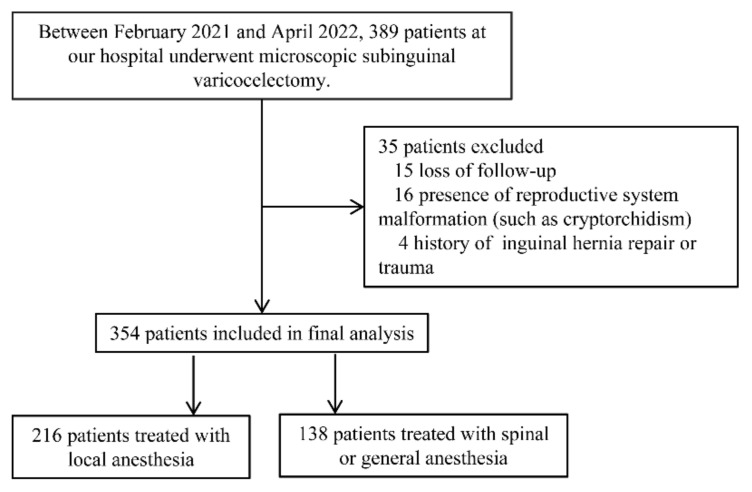
Flowchart of the study.

This prospective study was conducted at the Shengjing Hospital of China Medical University, from February 2021 to April 2022. A total of 389 consecutive patients who underwent subinguinal microscopic varicocelectomy were enrolled in the present study. After screening, there were 354 patients included in the final analysis; of these, 216 patients were treated under local anesthesia, and 138 patients were treated under general anesthesia or spinal anesthesia, respectively (Figure 1).

### 2.2. Inclusion and Exclusion Criteria

Inclusion criteria: age more than 18 years; clinically palpable varicocele; infertility with abnormal semen parameters (concentration, total number, motility, or morphology), nonobstructive azoospermia, symptomatic varicocele (intermittent or persistent sensation of dull pain or discomfort within the scrotal or groin region), American Society of Anesthesiologists (ASA) physical classification I or II.

Exclusion criteria: patients who had previous varicocelectomy (recurrent) or other types of inguinal surgery (such as hernia repair); history of substance abuse, chronic analgesic use, and allergies to local anesthetics; malformation of the reproductive system; the presence of lower urinary-tract infection or prostatitis, epididymitis, and seminal vesiculitis.

Decision-making process: patients were given a choice to undergo local anesthesia, spinal, or general anesthesia, after receiving counseling about the advantages and drawbacks of the procedures (such as effectiveness, costs, hospital duration, and complications). After receiving the explanation, the patients selected the method they preferred, and provided informed consent; their chosen procedure was performed by senior experts with vast experience in using these techniques.

### 2.3. The Technique of Subinguinal Microscopic Varicocelectomy under Local Anesthesia (LA)

**Figure 2:**
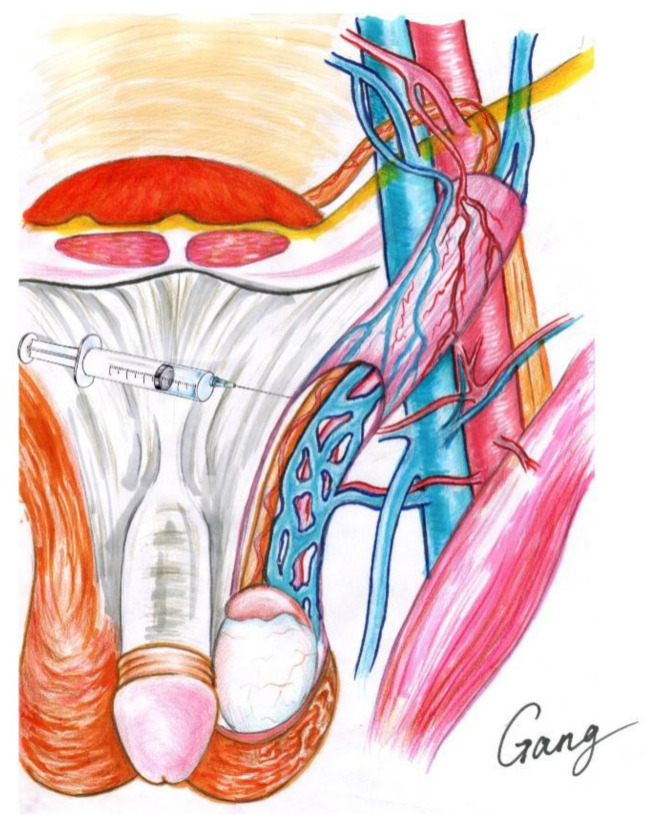
Local anesthesia of microscopic varicocelectomy.

Local anesthesia was delivered by infiltrating 2% lidocaine (10 mL) and 0.5% ropivacaine (20 mL). The patient was placed in a supine position. First, local infiltration into the incision site was applied layer by layer, and then an incision of about 2–3 cm was made below t the external inguinal ring. Second, after incising the skin and subcutaneous tissue and blocking the spermatic nerve (Figure 2), the spermatic cord was identified and then elevated, using gentle traction with an appendage forceps, and passed through the incision, facilitating the placement of the spermatic cord to the skin incision with a piece of rubber sheet. Third, all identifiable external spermatic veins were divided and ligated. The cremaster muscle was then opened. Fourth, the spermatic cord was separated into three parts (cremaster muscle, vas deferens, and other parts of the internal spermatic cord); the testis was not delivered, and the gubernacular veins were not handled. The spermatic vascular bundle was then placed under a microscope with a resolution increase of 10× to 20× magnification. Fifth, the internal spermatic veins were carefully separated, skeletonized, and ligated with nonabsorbable 4-0 silk threads; care was taken to preserve the testicular artery and lymph vessels; the testicular arteries were identified by their pulsation and blood flashing. Sixth, the spermatic cord was returned to its bed, if no bleeding was observed. The incision was closed layer by layer, with 3-0 absorbable sutures. The same method was used to treat the contralateral side. No sedation or other analgesics were administered intraoperatively.

On the day of admission, the patient could eat and drink freely, intraoperative monitoring of blood pressure and an electrocardiogram (ECG) was performed, and the nervousness and anxiety of the patient were relieved by a conversation with the doctor and by enjoying music. After surgery, patients were monitored for one hour and discharged if vital signs were stable.

### 2.4. The Protocol of General Anesthesia and Spinal Anesthesia (GA and SA)

All patients underwent inguinal varicocelectomy by general anesthesia induced with fentanyl (0.2–0.3 μg/kg), cisatracurium (0.15 mg/kg), and propofol (3–5 μg/mL). Patients were intubated and ventilated with 50% oxygen and 50% N_2_O. Anesthesia was maintained with propofol (target-controlled infusion 3–5 μg/mL) and remifentanil (continuous infusion 0.05–0.3 μg/kg/min). Anesthesia management was left to the attending anesthesiologist. No other analgesics were administered intraoperatively.

The patients in the SA group were preloaded with 10 mL/kg of isotonic lactated Ringer’s solution. They were administered SA using the midline approach with a 25-gauge Quincke needle at the L3–L4 or L2–L3 intervertebral space in the lateral decubitus position, with the operative side remaining dependent. The subarachnoid injection contained 2.5 mL of 5 mg/mL heavy bupivacaine (Marcaine 0.5% [5.0 mg/mL] spinal heavy). These blocks were administered by one of the anesthetists. After removing the spinal needle, the patients were placed in a horizontal position for surgery. The sensory block was assessed using the pinprick test, and surgery was allowed to begin when the sensory block was higher than T10. The motor block was evaluated using a modified Bromage scale.

### 2.5. Measurement of Characteristics and Follow-Up

Patient demographics (age and body mass index (BMI)), comorbidity (ASA classification), indications for varicocelectomy (infertility with poor semen parameters, scrotal pain or discomfort, and nonobstructive azoospermia (NOA)), varicocele data (surgical side (left vs. bilateral), grade [(eft, II vs. III)), semen parameters before MSV (total sperm number, sperm concentration, total motility, progressive motility, and morphology), intraoperative data (operating duration, and the number of ligated veins), perioperative visual analog scale (VAS) scores (intraoperative (record the highest VAS scores during surgery), 3 h and one day after surgery without analgesics, postoperative analgesic requirement), anesthesia-associated side effects (nausea or vomiting, headache or postspinal backache, urinary retention and hypotension), and cost data (satisfaction for patients, hospital stay, days to return to normal activity, and mean costs).

The diagnosis of **varicocele** was determined by the physical examination in the upright position. The VC was categorized based on the **Dubin and Amela grading system**: Grade 1: palpable during Valsalva maneuver. Grade 2: palpable at rest. Grade 3: visible and palpable at rest. The grading was determined by palpation by two surgeons independently. **Infertility** is the absence of a desired pregnancy following regular, unprotected sexual intercourse for at least one year [13]. **The analysis of semen parameters** was conducted according to the WHO laboratory manual for the examination and processing of human semen (5th edition). Patients received nonsteroidal anti-inflammatory (e.g., flurbiprofen 50 mg or ketorolac 30 mg by intravenous injection) or opioid drugs (pethidine 50 mg by intramuscular injection) postoperatively, for pain; for instance, the VAS score was more than 4, and the patient asked for pain relief; this was recorded as the **post analgesia requirement**. **Time to return to normal activity** was calculated by the time needed to perform the activities of daily living (such as dressing, bathing, feeding oneself, housework, and work, not including extraneous exercise) with pain. **Patients’ satisfaction** (good vs. bad) was based on their overall feeling concerning their comfort and activity at the end of the follow-up period [14].

**The primary outcome** was the evaluation of pain during and after surgery, which was determined using a visual analog scale (VAS, ranging from 0 (no pain) to 10 (worst) pain). Patients under LA were uniquely asked about pain or discomfort during the operation. Then, both groups assessed postoperative pain at predetermined time intervals (3 h and one day after surgery). **The secondary outcome** measures were postoperative analgesic requirement, the incidence of anesthesia-associated adverse effects, short-term postoperative surgical-related complications within one month (including injection-site pain/bruising, scrotal hematoma, etc.), re-admission rate within one month, and cost data.

**Postprocedural follow-up** visits were performed on the day of discharge, to assess the patients’ time to return to normal activity, the side effects, and satisfaction.

### 2.6. Sample Size Calculation

Type I error α was 0.05, type II error β was 0.10 [power (1 − β) = 0.90], the 2-tailed *p* value was <0.05, the study and control group ratio was 2:1, and at least 184 patients in the LA group and 92 patients in the GA or SA group were included, respectively. It was calculated by the PASS 11.0 (Power Analysis and Sample Size 11.0, NCSS Inc., Kaysville, UT, USA) [11].

### 2.7. Statistical Analysis

Data were analyzed by the SPSS 22.0 for Windows (SPSS Inc., Chicago, IL, USA). To determine the normality of continuous variables, the Kolmogorov–Smirnov test was applied. Continuous variables were presented as the median (interquartile range) or mean ± standard deviation. Categorical variables were reported as the numbers (percentage). Before PSM, independent samples were subjected to the Student’s *t*-test, to compare the mean of two continuous normally distributed variables. The Mann–Whitney U test was used to compare the mean of two continuous non-normally distributed variables. The χ^2^ test or Fisher’s exact test was used for categorical variables. After PSM, the categorical variables were compared using binary conditional logistic regression. Paired samples subjected to the Student’s *t*-test were used to compare the mean of two continuous normally distributed variables, and the Wilcoxon test was used to compare the mean of two continuous non-normally distributed variables, using univariate analysis. A *p*-value of less than 0.05 was considered statistically significant.

## 3. Results

**Figure 3:**
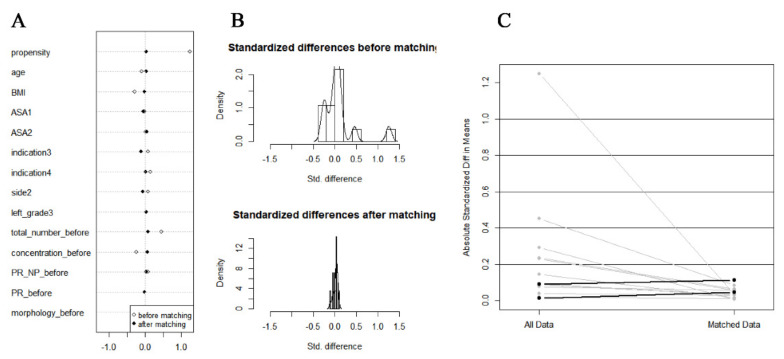
The plot of the propensity score-matched study. (**A**) Dot plot of standardized mean difference. (**B**) Histogram of standardized mean differences (before and after). (**C**) Line plot of individual differences.

We used the propensity score matching (PSM) method to adjust baseline confounding variables between the LA and GA or SA groups to derive more accurate conclusions. Multivariate logistic regression analysis was used to determine propensity scores for each patient based on age, BMI, ASA, surgical indications, varicocele data, and semen parameters before MSV (all baseline variables in Table 1). The LA and GA or SA groups were matched 2:1 using a caliper width 0.2 of the standard deviation of the logit of the propensity score, through the nearest neighbor matching (Figure 3).

There were 354 patients enrolled in the final analysis, divided into two groups. There were 216 patients treated with MSV under LA and 138 patients treated with MSV under GA or SA. The mean age of both groups was comparable (31.4 ± 4.9 vs. 31.0 ± 4.6, *p* = 0.432), and the mean BMI of patients in the LA group was lower compared with the patients in the control group (25.2 ± 3.4 vs. 26.2 ± 3.6, *p* = 0.011). After PSM, we balanced baseline variables between the two groups. Finally, there were 161 patients treated under LA and 102 patients treated under GA or SA, respectively (Table 1, Figure 3).

After PSM, we found that the operating duration was comparable between the LA and GA or SA groups for unilateral MSV and bilateral MSV (*p* = 0.617, *p* = 0.733). The number of ligated veins was also equivalent between the two groups. The VAS score for patients in the LA group during surgery was 1.01 ± 0.15, and no patient needed intraoperative conversion from LA to GA/SA. The patients in the GA or SA group suffered from higher VAS scores both three hours and one day after surgery; a higher incidence of postoperative analgesic requirement was observed in the GA or SA group (all *p* < 0.001), see details in Table 2.

A higher ratio of patients experienced anesthesia-related side effects in the GA or SA group than in the LA group, such as nausea or vomiting, headache or postspinal backache, urinary retention, and hypotension (*p* = 0.011, *p* = 0.001, *p* = 0.029, and *p* = 0.074, respectively). There was no patient who experienced either postoperative surgery-related complications or re-admission among these two groups. The rate of perioperative satisfaction for patients based on their overall feeling concerning comfort and activity was higher, and the hospital stay and days to return to normal activity were shorter in the LA group, compared with the patients in the GA or SA group (*p *= 0.029, *p* < 0.001, and *p* < 0.001, respectively). The patients in the LA group spent less money than the patients in the GA or SA group (*p* < 0.001); see details in Table 2.

## 4. Discussion

Acute postoperative pain is a common complication after inguinal surgery [15]. Patients frequently report acute postoperative pain after varicocelectomy, especially young males, who are more susceptible to pain than older patients [16]. Therefore, perioperative pain-management is significant in patients after MSV. In this prospective cohort study using PSM, we found that LA was associated with reduced perioperative pain scores and decreased postoperative analgesic requirements, compared with GA and SA. In addition, LA lowered the incidence of postoperative complications, and was accompanied by a higher rate of overall satisfaction for patients, a shortened hospital stay and fewer days to return to normal activity, and less cost.

We found that LA had an acceptable intraoperative pain score, and prominent advantages concerning postoperative pain relief and postoperative analgesic requirements, compared with GA or SA. Although many investigators have reported pain or discomfort at the operation site during hernia repair under LA [17], few of our patients felt the incision, and a significantly lower pain score was observed. Moreover, they did not experience any pain requiring additional analgesics and intraoperative conversion from LA to GA/SA. Consistent with our findings, Kadihasanoglu et al. [11] in an RCT study enrolled 60 patients who underwent subinguinal varicocelectomy, and indicated that the VAS of pain under local anesthesia (2% prilocaine) postoperatively was comparable with patients under SA. However, the mean dosage of injected diclofenac requirement was higher in the SA compared with LA groups. The possible reason may be that ilioinguinal, iliohypogastric, and genitofemoral nerves were blocked by long-acting local anesthetic, to reduce the discomfort and pain during the division and ligation of the spermatic cord, subsequently relieving the pain of the incision and scrotum after surgery. In addition, Hsu et al. [18], in a case-study series that enrolled 575 patients, also reported successful results for subinguinal varicocelectomy under LA, with a mixture of lidocaine and epinephrine. In their study, the pain level was assessed using a VAS, with a mean score of 1.8, which is similar to our outcome. It is worth noting that a recent study clearly showed that even under general anesthesia, the application of local anesthetics at the site of the varicocelectomy and skin incisions showed a significant reduction in pain level, compared with the patients who did not receive any local anesthetic during the surgery. At the same time, the study showed that levobupivacaine showed a significantly lower pain reduction at VAS than lidocaine [19].

On the contrary, a retrospective study that included 3565 patients with varicocele demonstrated that pain relief in patients treated with inguinal MSV under LA (2% lidocaine) was comparable to the patients treated with subinguinal MSV under GA or SA [10]. This discrepancy could be attributed to the different anesthetic and surgical regimens. Lidocaine is believed to hasten the onset of the block, and ropivacaine provides a prolonged duration of the sensory block extending into the postoperative period, which is an excellent mixture for LA. The perioperative VAS scores between the two groups were less than four; therefore, both anesthetic protocols were adequate for varicocelectomy.

No significant difference was found in the operating duration and the number of ligated veins between the two groups. This indicates that LA does not affect the operative process. Al-Said et al. [20] reported that the average operative time was 62 ± 17 min and 109 ± 19 min, for unilateral and bilateral MSV, respectively. This is a little longer than our results; the discrepancy might be due to differences in the practical microscopic skills of centers. We determined that operating duration is closely related to the number of ligated veins; furthermore, MSV requires much more practical skill than other techniques. Thus, many studies have reported longer operative times with the microscopic technique.

In this study, LA was also accompanied by fewer anesthesia-associated side effects than GA or SA in patients undergoing subinguinal MSV, such as nausea or vomiting, headache or postspinal backache, and urinary retention; these are known common side effects of GA or SA, which might delay postoperative recovery [15]. In addition, there was no patient who experienced either postoperative surgery-related complications or re-admission, among these two groups.

All patients in the LA group were discharged on the day of operation, meaning that it was day surgery, and they were able to achieve normal activity as soon as they returned to their rooms. Therefore, LA was superior to GA or SA in both hospital stay and days to return to normal activity. In line with this, Watanabe et al. [21], in a retrospective study with 144 infertile patients with varicocele, also indicated that the hospitalization period required for subinguinal MSV under LA was significantly shorter compared with high retroperitoneal ligation under SA or laparoscopic ligation under GA. Furthermore, excellent pain relief following LA allows patients to quickly return to normal daily activities and enhance their quality of life. Moreover, LA also frees the patients from fasting before surgery, with no need for intravenous infusion and catheterization after surgery, which further accelerates the time to return to normal life by a few hours [16]. As a result, the medical costs were also dramatically reduced by about 40%, and the patients’ medical experience could also be significantly improved.

Apart from the benefit mentioned above, there were other latent advantages. Firstly, LA could facilitate understanding of the nerve distribution in the spermatic cord by the intraoperative conversation between patient and surgeon. Therefore, it can avoid nerve injury and thus prevent postoperative chronic orchialgia. Secondly, this technique could also be used for overweight patients, because the subinguinal approach deals with a superficial site and avoids the need for opening the abdominal muscles and external oblique aponeurosis.

However, there are several potential risks. Firstly, short-term hospital stay may also lead to poor wound care and complications (such as hemorrhage or hematoma and infection) if the compliance is poor. Secondly, preoperative and intraoperative anxiety is common among all patients undergoing LA. A detailed explanation of the procedure should be provided before surgery, to address this problem. In additions, playing music and conversing with the patient throughout the procedure may also be helpful. The combined use of sedative drugs and anti-anxiety drugs is also beneficial. Thirdly, compared with LA, patients treated with GA or SA may have a lower risk of intra-operative movement, which is critical in preventing complications such as arterial injury or lymphatic injury. However, there was no such intra-operative movement or subsequent complications observed in this study. In addition, both the number of ligated veins and the duration of surgery were comparable between the two groups, indicating no limitation on executed surgery and operative time.

This study had several limitations. Firstly, the study was non-randomized, and from a single center. Secondly, we self-reported and established part of the postoperative complications and satisfaction level of patients. Thirdly, in other countries, men undergoing MSV may be treated as outpatients, limiting the generalizability of the outcomes measured in terms of hospital length of stay. Fourthly, fertility-related outcomes (change in semen parameters, or pregnancy rates, etc.) and surgery-related complications were not reported. Nevertheless, this is the first prospective PSM cohort study to explore pain relief, safety, and cost among LA and GA or SA in patients who underwent subinguinal MSV. Multi-center RCTs with fertility-related outcomes and long-term surgery-related complications are needed in the future, for confirming these results.

## 5. Conclusions

This prospective PSM cohort demonstrated that LA has the advantages of perioperative pain relief, reduced anesthesia-associated side effects, and cost compared with GA or SA. It indicated that LA is an effective and safe technique for subinguinal MSV and may guide clinical practice.

## Figures and Tables

**Table 1:** Demographics and clinical data in this cohort according to the anesthesia type before and after PSM.

	Propensity before 354 Patients			Propensity after 263 Patients		
Variables	GA or SA	LA	*p* Value	GA or SA	LA	*p* Value
Number of patients (%)	138 (100.00)	216 (100.00)		102 (100.00)	161 (100.00)	
**Demographic characteristics**						
Mean age (years)	31.4 ± 4.9	31.0 ± 4.6	0.432	31.4 ± 5.1	31.2 ± 5.7	0.725
BMI (kg/m^2^)	26.2 ± 3.6	25.2 ± 3.4	0.011	26.0 ± 3.6	25.5 ± 3.6	0.249
ASA (grade I vs. grade II)	132 (95.7)/6 (6.3)	206 (95.4)/10 (4.6)	0.901	97 (95.1)/5 (4.9)	153 (95.0)/8 (5.0)	0.981
**Surgical indications**			0.216			0.908
Infertility with poor semen parameters	123.00 (89.1)	178.00 (82.4)		89.00 (87.3)	143.00 (88.8)	
Scrotal pain or discomfort	7.00 (5.1)	16.00 (7.4)		7.00 (6.9)	9.00 (5.6)	
NOA	8.00 (5.8)	22.00 (10.2)		6.00 (5.9)	9.00 (5.6)	
**Varicocele data**						
MSV side (left vs. bilateral)	80 (58.0)/58 (42.3)	117 (54.2)/99 (45.8)	0.482	58 (56.9)/44 (43.1)	90 (55.9)/71 (44.1)	0.878
Grade (left, II vs. III)	64 (46.4)/74 (53.6)	96 (44.4)/120 (55.6)	0.722	49 (48.0)/53 (52.0)	73 (45.3)/88 (54.7)	0.669
**Semen parameters before MSV**	371.00 (53.00)	158.00 (48.90)	0.224	371.00 (53.00)	158.00 (48.90)	0.224
Total sperm number (10^6^/ejaculate)	48.3 ± 29.5	65.2 ± 37.3	<0.001	56.3 ± 27.9	62.7 ± 38.4	0.119
Sperm concentration (10^6^/mL)	31.4 ± 19.7	26.5 ± 20.7	0.030	25.8 ± 18.4	27.4 ± 22.2	0.552
Total motility (PR + NP, %)	17.6 ± 11.4	18.8 ± 13.5	0.387	17.8 ± 11.42	18.3 ± 13.2	0.757
Progressive motility (PR, %)	14.7 ± 9.1	14.4 ± 11.6	0.837	13.7 ± 9.2	14.0 ± 11.6	0.831
Morphology (normal forms, %)	3.04 (1.54, 4.16)	2.01 (1.03, 4.02)	0.013	2.70 (1.26, 4.12)	2.01 (1.00, 4.01)	0.633

Continuous variables were expressed as median (interquartile range) or mean ± standard deviation; categorical variables were reported as numbers (percentage). Before PSM, independent samples Student’s *t*-test was used to compare the mean of two continuous normally distributed variables, and the Mann–Whitney U test was run to determine the mean of two continuous non-normally distributed variables. After PSM, the categorical variables were compared by binary conditional logistic regression. Paired samples Student’s *t*-test was used to compare the mean of two continuous normally distributed variables, and the Wilcoxon test was used to compare the mean of two continuous non-normally distributed variables in univariate analysis. Abbreviations: PSM, propensity score-matching; GA, general anesthesia; SA, spinal anesthesia; LA, local anesthesia; BMI, body mass index; ASA, American Society of Anesthesiologists; NOA, nonobstructive azoospermia; MSV, subinguinal microscopic varicocelectomy; PR, progressive (a + b motility); NP = non-progressive.

**Table 2:** Perioperative data in this cohort according to the anesthesia type before and after PSM.

	Propensity before 354 Patients			Propensity after 263 Patients		
Variables	GA or SA	LA	*p* Value	GA or SA	LA	*p* Value
Number of patients (%)	138 (100.00)	216 (100.00)		102 (100.00)	161 (100.00)	
**Intraoperative data**						
Operating duration						
Unilateral MSV (minutes)	40.2 ± 9.3	40.1 ± 2.7	0.912	40.7 ± 9.4	40.1 ± 2.8	0.617
Bilateral MSV (minutes)	74.4 ± 8.0	75.2 ± 8.8	0.551	74.1 ± 7.7	74.7 ± 9.2	0.733
Number of ligated veins						
Unilateral MSV (number)	10.0 (8.0, 12.0)	9.0 (8.0, 12.0)	0.428	10.0 (8.0, 12.0)	9.0 (8.0, 12.0)	0.395
Bilateral MSV(number)	21.0 (17.8, 25.0)	20.0 (17.0, 25.0)	0.498	22.0 (18.0, 26.0)	21.0 (17.0, 25.0)	0.430
**Intraoperative conversion from LA to GA/SA**	N/A	0 (0.0)	N/A	N/A	0 (0.0)	N/A
**Perioperative VAS scores**						
VAS during surgery (scores)	N/A	1.02 ± 0.18	N/A	N/A	1.01 ± 0.15	N/A
VAS three hours after surgery (scores)	2.67 ± 1.51	1.00 ± 0.18	<0.001	2.59 ± 1.57	1.02 ± 0.14	**<0.001**
VAS one day after surgery (scores)	2.04 ± 1.27	1.00 ± 0.07	<0.001	2.04 ± 1.30	1.00 ± 0.00	**<0.001**
Postoperative analgesic requirement (yes)	14 (10.1)	0 (0.0)	<0.001	11 (10.8)	0 (0.0)	**<0.001**
**Anesthesia-associated side effects**						
Nausea or vomiting (yes)	9 (6.5)	0 (0.0)	<0.001	4 (3.9)	0 (0.0)	**0.011**
Headache or postspinal backache (yes)	8 (5.8)	0 (0.0)	<0.001	7 (6.9)	0 (0.0)	**0.001**
Urinary retention (yes)	3 (2.2)	0 (0.0)	0.030	3 (2.9)	0 (0.0)	**0.029**
Hypotension (yes)	2 (1.4)	0 (0.0)	0.076	2 (2.0)	0 (0.0)	0.074
**Postoperative surgery-related complications**	0 (0.0)	0 (0.0)	1.000	0 (0.0)	0 (0.0)	1.000
**Re-admission rate**	0 (0.0)	0 (0.0)	1.000	0 (0.0)	0 (0.0)	1.000
**Cost-effective data**						
Satisfaction for patients (good)	130 (94.2)	216 (100)	<0.001	99 (97.1)	161 (100)	**0.029**
Hospital stay (days)	2.08 ± 0.66	1.00 ± 0.00	<0.001	2.09 ± 0.69	1.00 ± 0.00	**<0.001**
Days to return to normal activity	1.49 ± 0.50	1.00 ± 0.00	<0.001	1.48 ± 0.50	1.00 ± 0.00	**<0.001**
Mean costs (Yuan/RMB) (days)	7680 ± 382	4644 ± 482	<0.001	7668 ± 380	4689 ± 462	**<0.001**

Continuous variables were expressed as median (interquartile range) or mean ± standard deviation; categorical variables were reported as numbers (percentage). Before PSM, independent samples Student’s *t*-test was used to compare the mean of two continuous normally distributed variables. The Mann–Whitney U test was run to determine the mean of two continuous non-normally distributed variables. After PSM, the categorical variables were compared by binary conditional logistic regression. Paired samples Student’s *t*-test was used to compare the mean of two continuous normally distributed variables. The Wilcoxon test was used to compare the mean of two continuous non-normally distributed variables in univariate analysis. Abbreviations: PSM, propensity score-matching; GA, general anesthesia; SA, spinal anesthesia; LA, local anesthesia; MSV, microscopic subinguinal varicocelectomy; VAS, visual analog scale; N/A, not applicable.

## Data Availability

Not applicable.

## References

[1] Damsgaard J., Joensen U.N., Carlsen E., Erenpreiss J., Jensen M.B., Matulevicius V., Zilaitiene B., Olesen I.A., Perheentupa A., Punab M. (2016). Varicocele Is Associated with Impaired Semen Quality and Reproductive Hormone Levels: A Study of 7035 Healthy Young Men from Six European Countries. Eur. Urol..

[2] Jukic M., Todoric M., Todoric J., Susnjar T., Pogorelic Z. (2019). Laparoscopic Versus Open High Ligation for Adolescent Varicocele: A 6-year Single Center Study. Indian Pediatr..

[3] Jensen C.F.S., Østergren P., Dupree J.M., Ohl D.A., Sønksen J., Fode M. (2017). Varicocele and male infertility. Nat. Rev. Urol..

[4] Pogorelić Z., Sopta M., Jukić M., Nevešćanin A., Jurić I., Furlan D. (2017). Laparoscopic Varicocelectomy Using Polymeric Ligating Clips and Its Effect on Semen Parameters in Pediatric Population with Symptomatic Varicocele: A 5-Year Single Surgeon Experience. J. Laparoendosc. Adv. Surg. Tech. A.

[5] Fallara G., Capogrosso P., Pozzi E., Belladelli F., Corsini C., Boeri L., Candela L., Schifano N., Dehò F., Castiglione F. (2022). The Effect of Varicocele Treatment on Fertility in Adults: A Systematic Review and Meta-analysis of Published Prospective Trials. Eur. Urol. Focus.

[6] Ding H., Tian J., Du W., Zhang L., Wang H., Wang Z. (2012). Open non-microsurgical, laparoscopic or open microsurgical varicocelectomy for male infertility: A meta-analysis of randomized controlled trials. BJU Int..

[7] Wang H., Ji Z.G. (2020). Microsurgery Versus Laparoscopic Surgery for Varicocele: A Meta-Analysis and Systematic Review of Randomized Controlled Trials. J. Investig. Surg..

[8] Bryniarski P., Taborowski P., Rajwa P., Kaletka Z., Życzkowski M., Paradysz A. (2017). The comparison of laparoscopic and microsurgical varicocoelectomy in infertile men with varicocoele on paternity rate 12 months after surgery: A prospective randomized controlled trial. Andrology.

[9] Hopps C.V., Lemer M.L., Schlegel P.N., Goldstein M. (2003). Intraoperative varicocele anatomy: A microscopic study of the inguinal versus subinguinal approach. J. Urol..

[10] Wang J., Liu Q., Wang X., Guan R., Li S., Zhang Y., Cheng Y., Zeng H., Tang Y., Zhu Z. (2018). Modified Inguinal Microscope-Assisted Varicocelectomy under Local Anesthesia: A Non-randomised Controlled Study of 3565 Cases. Sci. Rep..

[11] Kadihasanoglu M., Karaguzel E., Kacar C.K., Arıkan M.S., Yapici M.E., Türkmen N. (2012). Local or spinal anesthesia in subinguinal varicocelectomy: A prospective randomized trial. Urology.

[12] Moolagani V.R., Burla S.R., Neethipudi B.R., Upadhyayula S.M., Bikkina A., Arepalli N.R. (2019). Ropivacaine plus lidocaine versus bupivacaine plus lidocaine for peribulbar block in cataract surgery: A prospective, randomized, double-blind, single-center, comparative clinical study. J. Anaesthesiol. Clin. Pharmacol..

[13] WHO (2000). WHO Manual for the Standardized Investigation and Diagnosis of the Infertile Couple.

[14] Youssef T., Abdalla E. (2015). Single incision transumbilical laparoscopic varicocelectomy versus the conventional laparoscopic technique: A randomized clinical study. Int. J. Surg..

[15] Wu C.C., Bai C.H., Huang M.T., Wu C.H., Tam K.W. (2014). Local anesthetic infusion pump for pain management following open inguinal hernia repair: A meta-analysis. Int. J. Surg..

[16] Cui W.S., Shin Y.S., You J.H., Doo A.R., Soni K.K., Park J.K. (2017). Efficacy and safety of 0.75% ropivacaine instillation into subinguinal wound in patients after bilateral microsurgical varicocelectomy: A bi-center, randomized, double-blind, placebo-controlled trial. J. Pain Res..

[17] Olsen J.H.H., Laursen J., Rosenberg J. (2022). Limited use of local anesthesia for open inguinal hernia repair: A qualitative study. Hernia.

[18] Hsu G.L., Ling P.Y., Hsieh C.H., Wang C.J., Chen C.W., Wen H.S., Huang H.M., Einhorn E.F., Tseng G.F. (2005). Outpatient varicocelectomy performed under local anesthesia. Asian J. Androl..

[19] Pogorelić Z., Gaberc T., Jukić M., Tintor G., Nevešćanin Biliškov A., Mrklić I., Jerončić A. (2021). The Effect of Subcutaneous and Intraperitoneal Instillation of Local Anesthetics on Postoperative Pain after Laparoscopic Varicocelectomy: A Randomized Controlled Trial. Children.

[20] Al-Said S., Al-Naimi A., Al-Ansari A., Younis N., Shamsodini A., Khalid A., Shokeir A.A. (2008). Varicocelectomy for male infertility: A comparative study of open, laparoscopic and microsurgical approaches. J. Urol..

[21] Watanabe M., Nagai A., Kusumi N., Tsuboi H., Nasu Y., Kumon H. (2005). Minimal invasiveness and effectivity of subinguinal microscopic varicocelectomy: A comparative study with retroperitoneal high and laparoscopic approaches. Int. J. Urol..

